# Residual Characteristics and Processing Factors of Flubendiamide and Tebufenozide in Rice and Its Processed Products

**DOI:** 10.3390/foods14172925

**Published:** 2025-08-22

**Authors:** Dongju Kim, Eunbeen Oh, Seunghyeon Jo, Hyeonwoo Shin, Youngjin Ham, Junyoung Kim, Mihyun Cho, Moohyeog Im, Keesung Kyung

**Affiliations:** 1 Department of Environmental and Biological Chemistry, College of Agriculture, Life and Environment Sciences, Chungbuk National University, Cheongju 28644, Republic of Korea; kimdj6746@naver.com (D.K.); gsw06059@naver.com (E.O.); 2 Analytical Research Group, Translational Toxicology Research Division, Korea Institute of Toxicology, Jeongeup 56212, Republic of Korea; seunghyeon.jo@kitox.re.kr; 3 Residue GLP Research Team, Research Institute, NongHyup Chemical, Okcheon 29008, Republic of Korea; hyeonwoo@nhchemical.com; 4 Residue Study Division, AB Solution, Hwaseong 18514, Republic of Korea; youngjin0223@naver.com; 5 Regulation Team, Development & Marketing Department, Sungbo, Seoul 06174, Republic of Korea; tim8518@sbcc.kr; 6 Department of Food Engineering, College of Engineering, Daegu University, Gyeongsan 38453, Republic of Korea; c_mh97@naver.com (M.C.); imh0119@daegu.ac.kr (M.I.)

**Keywords:** pesticide residue, rice, risk assessment, processing, LC-MS/MS

## Abstract

This study aimed to evaluate residue changes in flubendiamide and tebufenozide during the processing of whole grain into milled rice, cooked rice, and rice cake, and to calculate their processing factors (PFs). For the processing study, pesticides were applied at three times the recommended rate based on Korea’s good agricultural practice (GAP), and processed products were prepared using conventional methods. Residual pesticide analysis was performed using a modified QuEChERS method and LC-MS/MS. The residue analysis method was validated based on parameters including LOQ, linearity, and accuracy at the LOQ, 10LOQ, and MRL levels, with the LOQ set at 0.01 mg/kg for all samples. During milling, which removes the hull, more than 90% of the pesticide residues were eliminated. Additional reductions exceeding 50% were observed during cooking and rice cake processing. All PFs, except for those in the hulls, were less than 1, indicating that processing reduces pesticide levels. Despite the use of threefold the GAP rate, the %ADI values for all processed products remained below 1%, suggesting negligible dietary risk. These findings provide scientific evidence supporting the safety of processed rice products regarding pesticide residues and highlight the importance of considering processing effects in dietary exposure assessments.

## 1. Introduction

Pesticides are generally recognized to play a crucial role in agricultural development because they can protect crops from various pests and weeds, reducing losses and improving the quantity and quality of food [1]. Without the use of pesticides, the harvest of fruits, vegetables, and cereals can be reduced by 78, 54, and 32%, respectively, due to pest injury [2]. However, pesticides are toxic organic compounds, and because the mode of action of each pesticide varies, they can pose a risk to humans and wild animals, including non-target species, when accidentally exposed [3]. Most countries establish pre-harvest intervals (PHIs) and maximum residue limits (MRLs) for pesticides used in crop production [4]. In the absence of established MRLs, Korea, Japan, and the European Union implement a positive list system and manage it at less than 0.01 mg/kg [5], and the United States implements zero-tolerance and manages it as a non-detection principle [6].

Pesticide residues in crops are influenced by various factors, including the dilution effect due to crop growth, where sunlight photolysis and growth dilution play pivotal roles [7]. The concentration of the pesticide spray solution significantly impacts the residue levels, affected by application methods and environmental conditions [8]. The physicochemical properties of the pesticides, such as water solubility and vapor pressure, determine their degradation and persistence on plant surfaces [9]. Environmental factors like temperature, humidity, and sunlight are crucial in pesticide degradation and physical loss [10]. Additionally, the morphological aspects of the crop, such as leaf area and surface characteristics, significantly affect the distribution and persistence of pesticide residues [11].

Rice is a staple food and an important agricultural commodity in many countries. In addition to domestic consumption, it is also exported to various countries, including those in Southeast Asia and the United States, highlighting its global market importance. Also, because each country has different pesticide use patterns, MRLs may be set differently or may not be established at all in different countries. In Korea, the MRLs for flubendiamide and tebufenozide in rice have been established by the Ministry of Food and Drug Safety (MFDS) at 0.5 and 0.3 mg/kg, respectively. However, in the United States, there are no MRLs for these pesticides in rice, and therefore, a zero-tolerance policy is applied [12,13]. Therefore, the use of such pesticides in Korea may lead to refusal due to the presence of residual pesticides during customs clearance for the United States, which presents a significant obstacle to trade between Korea and the United States. These problems are not limited to this case and can also be applied to other crops imported from different countries. In the United States, MRLs are established based on the residue levels in whole grain, while in Korea, MRLs are set based on the residue concentrations in husked rice. Therefore, when considering actual consumption forms, the risk assessment based on residue levels may be overestimated.

When consuming agricultural products, raw materials are rarely consumed immediately, and most of them are washed with water [14]. Processing stages may reduce pesticide residues on agricultural products, although complete removal is not always achieved. In the case of non-systemic pesticides, most residual pesticides can be removed in the peeling process because they do not translocate into the inner parts of the agricultural products [14,15]. In addition, pesticide residues can be reduced through cooking processes at a high temperature or high pressure [16]. However, the dissipation pattern of pesticides can vary depending on their physicochemical properties [17].

Rice (*Oryza sativa* L.), a staple food for a significant portion of the world’s population, is rich in carbohydrates, fats, fibers, proteins, vitamins, minerals, and various bioactive compounds. It is known for its health benefits, including antioxidant, anticancer, antidiabetic, and anti-inflammatory activities [18]. In Korea, rice is cultivated nationwide and is consumed as a staple food. Rice is not directly consumed in the form of harvested whole grains, but is processed into husked rice and polished rice using a rice polishing machine. It is then further processed into cooked rice and rice cake for consumption. Therefore, it is necessary to elucidate residue patterns not only in whole grains but also in processed products. In the case of a processing study, if the residue levels of pesticides in processed products are less than the limit of quantification (LOQ), it is not possible to calculate processing factors. According to the FAO manual, in the case of a processing study, an exaggerated application rate is required to obtain sufficiently high residue levels [19].

Research on processing factors that indicate the increase or decrease of pesticide residues in processed agricultural products is important. However, research focusing on rice processing has been insufficient. Therefore, this study aims to elucidate the changes in pesticide residues during the processing of rice into various processed products and to provide clear evidence on pesticide residues in rice products consumed in the United States, Europe, and Asia, thereby ensuring consumer safety.

## 2. Materials and Methods

### 2.1. Field Trials

The rice used in this study was Samkwang, a variety of *Oryza sativa* L. Pesticide was sprayed in a test field in Anseong, Gyeonggi Province (Republic of Korea). In the processing study, the area of the control plot was 80.0 m^2^ (2.0 m × 40.0 m), while the areas treated with the test pesticides, flubendiamide and tebufenozide, were 144 m^2^ (2.0 m × 72.0 m). Each treatment plot consisted of three replicates. The commercial products of the pesticides used in the study were a mixture of flubendiamide 4% and clothianidin 2.0% suspension concentrate (SC), and tebufenozide 8% wettable powder (WP). The tested pesticides, flubendiamide and tebufenozide, were diluted 2000/3 times and 1000/3 times, respectively, and then sprayed onto the rice crops under immature conditions using a backpack sprayer. Flubendiamide was applied up to 14 days before harvest, and tebufenozide was applied up to 7 days before harvest, with each being applied at maximum three times in 10-day intervals. A processing study was conducted using a rate that was three-times higher than the application rate based on the Korean GAP to increase the probability of detecting residues in processed products. The contents of the active ingredient and the application rate per treatment for each test pesticide are presented in Table 1.

For the processing study, whole grains weighing 74.6–83.6 kg and 79.5–80.7 kg were harvested from flubendiamide-treated and tebufenozide-treated plots, respectively, while 40.2–42.2 kg were collected from the control plots. After harvest, whole grain samples from the processing study were transported to the greenhouse within 24 h and dried for 7 days until the water content was less than 15%. After drying, whole grain was processed into its various products, including husked rice, polished rice, hulls, husked rice and polished rice cooked in a pressure cooker, husked rice and polished rice cooked in an electric cooker, and rice cake. The processed samples were individually packed into self-sealing plastic bags and stored frozen at −20 °C until analysis.

### 2.2. Processing Procedures and Sample Preparation

**Figure 1:**
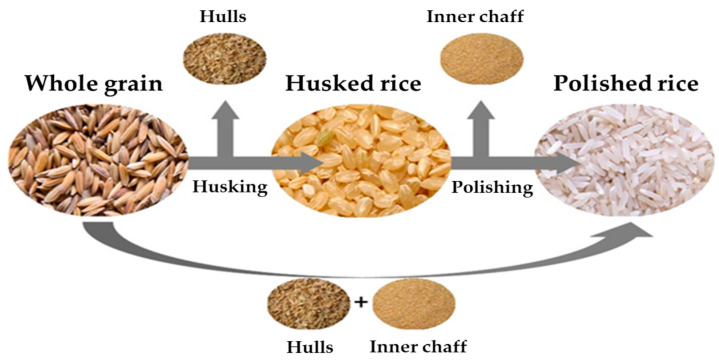
The milling process from whole grain to husked rice and polished rice.

Whole grain was milled to produce husked rice and polished rice using a rice milling machine (DY-5000R, Dongyang Comprehensive Machine, Yeongcheon, Republic of Korea). As shown in Figure 1, when processing from whole grain to husked rice, hulls are produced, and when processing from whole grain to polished rice, both hulls and inner chaff are produced. These samples were individually packed in self-sealing plastic bags and stored at −20 °C until analysis. The rice cooking method, using either a pressure cooker or an electric cooker, followed the cooking practices of ordinary households and restaurants. The rice cooking procedure detailed for both a pressure cooker and an electric cooker involves washing husked and polished rice three times with a water volume 1.5 times that of the rice. For the pressure rice cooker (CRP-HD1010FI, Cuckoo, Yangsan, Republic of Korea), husked rice is then soaked for 30 min, drained, and cooked with fresh water equal to 1.5 times its weight for 30 min; polished rice follows the same process but is cooked for only 20 min. In the electric rice cooker (SB-56RC, Kitchen Art, Incheon, Republic of Korea), the same pre-cooking steps are applied to husked rice, which is then cooked for 30 min, and to polished rice, which is cooked for 40 min.

Rice cake was prepared following commercial methods used by the manufacturing company. Polished rice (1.7 kg) was washed three times with water, immersed in water for 12 h, then drained. Salt (1%) was added to the rice before grinding it using a rice grinder (Duksan Food Co., Ltd., Chungbuk, Republic of Korea). The ground powder was sieved through a 20-mesh screen, homogenized, mixed with 10% water and 10% sugar, then kneaded. The dough was steamed at 100 °C for 20 min using a steamer (HY-2009-A, Hanyang Metal, Ulsan, Republic of Korea) and then cooled to room temperature. The processed samples were individually packed into self-sealing plastic bags and stored frozen at −20 °C until analysis.

### 2.3. Reagents and Materials

Analytical-grade flubendiamide (99.4% purity) and tebufenozide (100.0% purity) were obtained from Sigma-Aldrich (Saint Louis, MO, USA). Acetonitrile (HPLC grade) for standard dilutions, sample extraction, and the LC mobile phase was obtained from Burdick & Jackson (Muskegon, MI, USA). Additionally, HPLC-grade water and methanol for the LC mobile phase were also obtained from Burdick & Jackson (Muskegon, MI, USA). To enhance the sensitivity of the signals and the stability of retention times during analysis, formic acid (>98%) and ammonium formate used in the LC mobile phase were sourced from Samchun (Seoul, Republic of Korea) and Kanto Chemical (Tokyo, Japan), respectively. The QuEChERS extraction packets were obtained from Phenomenex Kinetex^®^ (Torrance, CA, USA). Combi-514R (Hanil Scientific Inc., Gimpo, Republic of Korea) centrifuges were used. A 2010 Geno/Grinder^®^ automated homogenizer (SPEX SamplePrep LLC., Metuchen, NJ, USA) was used for sample extraction.

### 2.4. Sample Preparation

#### 2.4.1. Extraction

##### Whole Grain, Husked Rice, Polished Rice, and Cooked Husked Rice, Polished Rice, and Rice Cake

Each 10 g aliquot of the sample was weighed into a 50 mL conical tube and wetted with 10 mL of distilled water containing 1 g of NaCl for 1 h. After 10 mL of acetonitrile was added to the tube, the tube was capped and shaken at 1300 rpm for 5 min. Four grams of MgSO_4_, 1 g of NaCl, 1 g of trisodium citrate dihydrate and 0.5 g of disodium citrate sesquihydrate were added to the tube, which was shaken again at 1300 rpm for 1 min, followed by centrifugation at 4000 rpm for 5 min. After mixing 500 μL of the supernatant with 500 μL of acetonitrile to prepare the matrix-matched sample, the mixed solution was filtered using PTFE (0.2 µm) for LC-MS/MS analysis.

##### Hulls and Inner Chaff

A 10 g aliquot of the hulls and inner chaff was weighed into a 50 mL conical tube and then wetted with 15 mL of distilled water containing 1.5 g of NaCl for 1 h. After 20 mL of acetonitrile had been added to the tube, the tube was capped and shaken at 1300 rpm for 5 min. Four grams of MgSO_4_, 1 g of NaCl, 1 g of trisodium citrate dihydrate, and 0.5 g of disodium citrate sesquihydrate were added to the tube, which was shaken again at 1300 rpm for 1 min, followed by centrifugation at 4000 rpm for 5 min. After mixing 500 μL of the supernatant with 500 μL of acetonitrile to prepare the matrix-matched sample, the mixed solution was filtered using PTFE (0.2 µm) for LC-MS/MS analysis.

##### Straw

**Figure 2:**
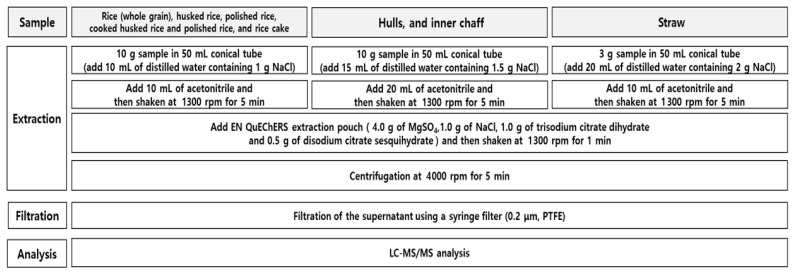
The QuEChERS method for the analysis of flubendiamide and tebufenozide residues in rice and its processed products.

A 3 g aliquot of the straw was weighed into a 50 mL conical tube and then wetted with 20 mL of distilled water containing 2.0 g of NaCl for 1 h. After 10 mL of acetonitrile had been added to the tube, the tube was capped and shaken at 1300 rpm for 5 min. Four grams of MgSO_4_, 1 g of NaCl, 1 g of trisodium citrate dihydrate, and 0.5 g of disodium citrate sesquihydrate were added to the tube, which was shaken again at 1300 rpm for 1 min, followed by centrifugation at 4000 rpm for 5 min. The standard solutions were diluted again with the control to make the solutions; the mixed solution was filtered using PTFE (0.2 µm) for LC-MS/MS analysis. The overall sample preparation procedure for all matrices is illustrated in Figure 2.

### 2.5. Instrumental Analysis

The tested pesticides, flubendiamide and tebufenozide, were analyzed using LC-MS/MS (Nexera X3 equipped with LCMS-8050, Shimadzu Corporation, Kyoto, Japan). The column for the analysis of flubendiamide and tebufenozide in the sample was Phenomenex Kinetex^®^ C18 (2.1 mm I.D. × 150 mm L., 2.6 μm particle size). Flubendiamide and tebufenozide were analyzed using an isocratic elution method. For the analysis of flubendiamide, a mixed solvent with a ratio of 20:80 (*v/v*) of distilled water containing 0.1% formic acid and acetonitrile containing 0.1% formic acid was used. Additionally, tebufenozide was analyzed as a mixed solvent with a ratio of 20:80 (*v/v*) of distilled water and methanol. Both mobile phases were supplemented with 10 mM of ammonium formate and 0.1% formic acid. The flow rate was 0.3 mL/min and the injection volume was 1 μL. The column temperature was maintained at 40 °C.

Mass spectrometry analysis was carried out using a triple-quadruple spectrometer LCMS-8050 (Shimadzu Corporation, Kyoto, Japan) equipped with an electrospray ionization (ESI) source, and flubendiamide and tebufenozide were analyzed in negative mode and positive mode, respectively. The MS conditions were typically as follows: ion spray voltage was 3.0 kV for flubendiamide, and 4.0 kV for tebufenozide; ESI source temperature was 150 °C for flubendiamide, and 300 °C for tebufenozide; Heat block temperature was 400 °C; and desolvation temperature was 250 °C; the collision gas was argon. The flow rates of the drying gas, heating gas, and nebulizing gas were 10 L/min, 10 L/min, and 3 L/min, respectively. Table 2 provides detailed MRM conditions of the target pesticide for instrumental analysis.

### 2.6. Method Validation

Validation of the optimized analytical methods was carried out in accordance with the guidelines described in the CODEX guidelines (CAC/GL-71) [20], containing accuracy, precision, linearity, the limit of detection (LOD), the limit of quantification (LOQ), matrix effects, and selectivity. The LOD was determined as the lowest concentration providing a signal-to-noise (S/N) ratio of 3, while the LOQ was determined as the lowest concentration providing an S/N ratio of 10 on the chromatogram. Pesticide extraction efficiencies were expressed in terms of the corresponding recoveries, which were calculated as shown in Equation (1). The recovery was evaluated at three fortification levels, LOQ, 10 LOQ, and the Korean MRL, with each level repeated five times. Accuracy was calculated as the average of the recovery (*n* = 5) at each fortification level, and precision was assessed in terms of its relative standard deviation (RSD). In the analysis of crop samples using LC-MS/MS, the matrix effect can influence quantification outcomes. The matrix effect refers to changes in the analytical signal due to both the composition of the sample matrix and the presence of impurities that co-elute with the target analyte [21]. Matrix-matched standards were used as part of the analysis process to ensure a more accurate quantification of the target analytes in the sample being analyzed. The linearity of the calibration curves was evaluated using the coefficient of determination (R^2^).

(1) $$Recovery\;(\%)=\frac{Amount\;of\;Pesticide\;treated}{Recoverd\;pesticide}\times 100$$

### 2.7. Processing Factors

The processing factor (PF) is used to determine the effect of processing on residue levels in a product [22]. The PF is affected by the yield of the processed product, and its value changes accordingly. Processing studies allow for the calculation of the processing factor for both the reduction and concentration of residues in processed products [19]. If the residue amount in the raw material is less than the LOQ, the PF is not calculated. A PF value more than 1 indicates an increase in residue amount during processing, while a PF value less than 1 indicates a decrease [23]. The PF for pesticide residues in food is defined as the ratio of the pesticide residue remaining in the food after processing to the pesticide residue amount in the raw material, reflecting the impact of the processing on the amounts of pesticide residues [24]. In this study, the PF for rice products was calculated according to the following formula:

(2) $$PF=\frac{Residue\;concentration\;(mg/kg)\;in\;processed\;product}{Residue\;concentration\;(mg/kg)\;in\;raw\;agricultural\;commodity}$$

### 2.8. Statistical Analysis

Welch’s ANOVA and *t*-test were conducted using Python 3.10.12 in this study. Data normality was first assessed using the Shapiro–Wilk test, and homogeneity of variance was evaluated using Levene’s test. Since the assumption of homogeneity was rejected (*p* < 0.05), indicating unequal variances, Welch’s ANOVA, which is appropriate for handling variance heterogeneity, was applied. The Games–Howell post hoc test, suitable for small sample sizes, was applied following Welch’s ANOVA [25]. This approach was used to evaluate the significant differences in pesticide residues across different crop parts and processing stages (*p* < 0.05). On the other hand, *t*-tests were used to ascertain significant differences between the two groups. Specifically, the processing factors of tebufenozide and flubendiamide in processed products were compared to their pre-processing levels using a *t*-test, confirming statistically significant differences (*p* < 0.05).

## 3. Results and Discussion

### 3.1. Validation of the Analytical Method

**Figure 3:**
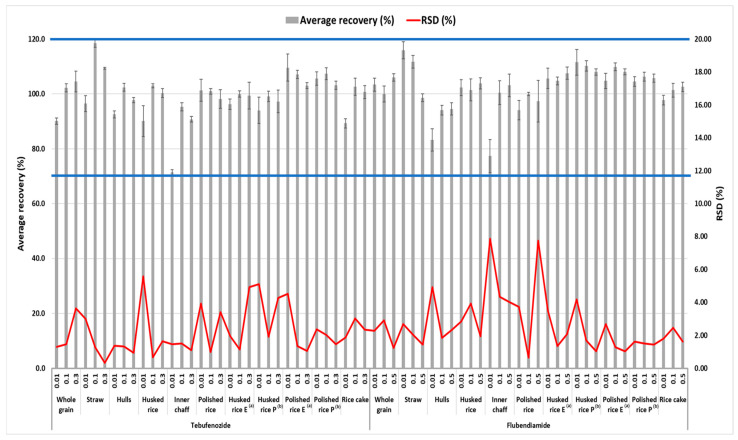
Recovery and RSD of test pesticides in straw and whole grain and its processed products for the validation of the analysis method. The effective range of the recovery is indicated by the blue line. In Figure 3, ^(a)^ and ^(b)^ mean processed rice cooked in an electric cooker and a pressure cooker, respectively.

**Figure 4:**
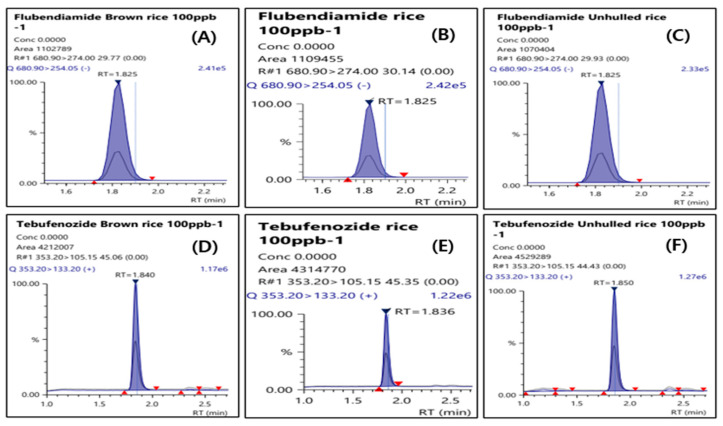
Chromatograms of matrix-matched standard at 100 µg L^−1^; flubendiamide (**A**) and tebufenozide (**D**) in husked rice, flubendiamide (**B**) and tebufenozide (**E**) in polished rice, and flubendiamide (**C**) and tebufenozide (**F**) in whole grain.

The LODs of flubendiamide and tebufenozide were 0.003 mg/kg for straw and 0.004 mg/kg for all other samples. The LOQs of these pesticides in the analyte were 0.01 mg/kg with an S/N ratio of 10 or greater. The linearities of the calibration curve for the analyses of the test pesticides in straw, whole grain, husked and polished grain, and their processed products including rice cakes and cooked rice, expressed as the correlation of determination (R^2^), were greater than 0.999 within the range of 2–100 µg/L (1–100 µg/L for straw, hull, and inner chaff), all of which complied with CAC-GL-71 [20]. Table 3 presents the summarized results. A recovery test was conducted at the LOQ, 10LOQ, and Korea’s MRL in husked rice to validate the analysis method for pesticide residue, and the results are presented in Figure 3. When recovery tests were conducted at the LOQ, 10xLOQ, and Korea’s MRL for straw, whole grain, husked and polished grain and their processed products including rice cakes and cooked rice to validate the analysis method for pesticide residue, the average recoveries and relative standard deviations (RSDs) of flubendiamide at all fortification concentrations in all samples ranged from 77.3% to 116.0% and from 0.66% to 7.86%, respectively. For tebufenozide, the average recovery and RSD for all fortification levels in the samples ranged from 71.4% to 118.5% and from 0.34% to 5.59%, respectively. Consequently, the results of the recovery tests conducted in this study met the criteria specified in CAC/GL 71 in all cases [20]. In addition, representative MRM chromatograms for husked rice, polished rice, and whole grain at a standard concentration of 100 µg/L for flubendiamide and tebufenozide (total of six chromatograms) are presented in Figure 4.

### 3.2. Results of Pesticide Residue Analysis

#### 3.2.1. Residual Concentration of Pesticide Whole Grain and Processed Products

**Figure 5:**
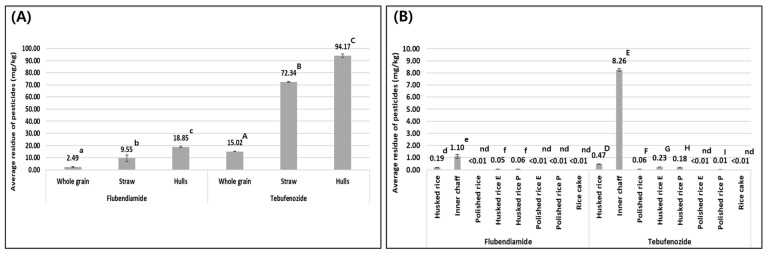
Mean residues of flubendiamide and tebufenozide (**A**) in whole grain, straw, and hulls, and (**B**) in husked rice, polished rice and its processed products; different letters in (**A**,**B**) indicate significant differences according to Welch’s ANOVA followed by the Games–Howell test (*p* < 0.05). The superscripts were denoted in lowercase for flubendiamide and in uppercase for tebufenozide. ND (not detected) means that the residue concentrations in processed products are less than the limit of quantification, making it impossible to compare differences in average residue concentrations.

Both test pesticides showed a general decrease in residue concentrations when whole grain was milled into husked and polished rice, while by-products of the milling process, such as hulls, showed increased concentrations of residues. The residue concentration of flubendiamide in whole grain was 2.49 mg/kg, and for hulls, inner chaff, husked rice, and polished rice, the concentrations were 18.85, 1.10, 0.19 mg/kg, and less than the LOQ, respectively. Similarly, the residue concentration of tebufenozide in whole grain was 15.02 mg/kg, and for hulls, inner chaff, husked rice, and polished rice, the concentrations were 94.17, 8.26, 0.47, and 0.06 mg/kg, respectively. The residue concentrations are shown in Figure 5.

This indicates that both non-systemic pesticides, flubendiamide and tebufenozide, primarily remain on the surface of the whole grain after application, and that most of the pesticide is removed during the milling process. The hulls, since they are lighter in weight than the whole grain, show a higher concentration of pesticide residues on a weight basis. This is similar to results of Giacinti et al. (2016), who reported that after treating apples with boscalid and ten other pesticides, more than 90% of all pesticide residues were found in the apple peel, with the highest residue concentrations observed in the peel, which is the lightest part of the fruit and where pesticides primary adhere [26]. The difference in residue concentrations between the two pesticide trials is attributed to variations in the active ingredient content at the time of application and the physicochemical properties of each pesticide. Indirect factors influencing residue levels include rain-fastness, foliar uptake, uptake through roots, volatilization, photostability, the degradation rate, and the persistence on plant surfaces, all of which are linked to the physicochemical properties of the active ingredients [27].

Inner chaff contains 20.85% fat, while husked rice contains 2.68–2.92%, and polished rice contains 0.21–0.66% [28]. The octanol–water partition coefficients (Log P) of flubendiamide and tebufenozide are 4.14 and 4.25, respectively, showing higher residue concentrations in inner chaff compared to husked and polished rice. It has been suggested that non-polar insecticides with a high Log P preferentially adhere to inner chaff, which has a higher fat content [29].

After milling rice into husked and polished rice and subsequent washing, changes in pesticide residue concentrations were observed during the cooking process using a pressure cooker for husked rice and an electric rice cooker for polished rice.

When cooking husked rice treated with flubendiamide using both a pressure cooker and an electric rice cooker, the residue concentrations of flubendiamide in the cooked husked rice were 0.06 and 0.05 mg/kg, respectively, with no statistically significant difference between the two cooking methods. This confirmed that the evaporation rate of flubendiamide, which has a vapor pressure of 0.1 MPa, acted similarly during cooking in both the pressure cooker and the electric rice cooker, and that there was no significant difference in the reduction in flubendiamide residue between high-pressure and normal-pressure cooking methods. Additionally, the residue concentration of flubendiamide in polished rice was already less than the LOQ before cooking, and all residue concentrations in the cooked polished rice were also less than the LOQ, indicating that a significant amount of flubendiamide had been removed during the milling process. Therefore, it was difficult to distinguish any difference in residue levels between the two cooking methods. Tebufenozide, with a vapor pressure of 1.56 × 10^−4^ MPa and a degradation point of 200 °C, was expected to degrade more rapidly under high temperature and pressure conditions. The study found that the residue concentration of tebufenozide in husked rice cooked in a pressure cooker was 0.18 mg/kg, lower than the 0.23 mg/kg residue concentration in husked rice cooked in an electric rice cooker. This indicates that the high temperature and pressure conditions of the pressure cooker contributed to the reduction in tebufenozide residue concentrations. Research investigating the effects of commercial and home processing on the removal of chlorpyrifos and carbosulfan residues from rice and the formation of metabolites during processing showed that residue reduction was more pronounced when cooking under high pressure than atmospheric pressure. It was concluded that high-pressure cooking methods are the most effective way to remove pesticide residues from rice and reduce the risk of dietary exposure [30]. In the case of polished rice, the residue concentrations of tebufenozide were low in both the pressure cooker and the electric rice cooker, showing concentrations of 0.01 mg/kg and less than 0.01 mg/kg (below the LOQ), respectively, making it difficult to identify a significant difference in residue concentrations between the two cooking methods. This is likely because most of the pesticide had already been removed during the milling process, resulting in no significant difference in residue concentrations depending on the cooking method.

In processed rice cakes, the residue concentrations of both flubendiamide and tebufenozide were less than the LOQ. The processing steps, including milling into polished rice, washing the rice, immersing for 12 h, grinding, and steaming, led to the removal, dissolution, and degradation of pesticides from the rice surface, resulting in reduced pesticide residue concentrations. Lee and Im (2021) investigated the changes in residue concentrations of etofenprox in processed rice cakes and cookies, finding that etofenprox was not detected in polished rice processed into rice cakes or cookies [31]. By using etofenprox immersed husked rice to study the residual characteristics during rice processing, it was found that the residue concentrations of etofenprox decreased by 78.0–90.2% during the processing into rice cakes and cookies [31]. This confirms that pesticides remaining on the surface are sufficiently removed through the processing steps, even when the test pesticides are applied at doses exceeding the safe usage standards. This is similar to the results of Zhang et al. (2011), where chlorpyrifos applied to rice mainly residues in rice straw and hulls [32]. 

The residue concentrations of flubendiamide and tebufenozide in straw were high, at 9.55 and 72.34 mg/kg, respectively. While straw is not consumed by humans, in many regions, fresh or processed straw and husk are used as feed for ruminant animals, and the persistence of pesticide residues induces a potential risk [32]. When using straw as livestock feed, caution regarding pesticide residues is necessary, and the consideration of pesticide use management and straw processing methods is deemed essential [33].

#### 3.2.2. Processing Factor

**Figure 6:**
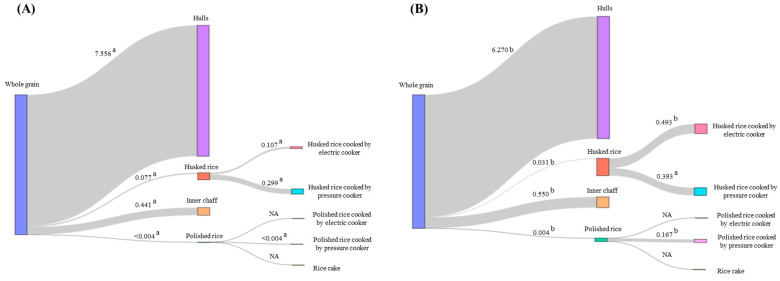
Sankey diagrams comparing the processing factors of the processed products for flubendiamide (**A**) and tebufenozide (**B**). The different letters between (**A**,**B**) in processing factors indicate a significant difference in *t*-test (*p* < 0.05). NA (not analyzed) indicates that the residue concentrations in the processed products are less than limit of quantification, making it impossible to calculate processing factors and compare differences between pesticides.

The processing factors for flubendiamide and tebufenozide are represented in a Sankey diagram in Figure 6. When considering the residue concentrates after processing for hulls, husked rice, inner chaff, and polished rice, the pre-processing residue concentrate corresponds to that of whole grain. For husked rice cooked by an electric cooker and husked rice cooked by a pressure cooker, the pre-processing residue concentrate corresponds to that of husked rice. For polished rice cooked by an electric cooker, polished rice cooked by a pressure cooker, and rice cake, the pre-processing residue concentrate corresponds to that of polished rice. The average processing factors for hulls of flubendiamide and tebufenozide were 7.56 and 6.27, respectively. These values represent the outer hulls generated when whole grain is processed into husked rice or polished rice. Since both pesticides are non-systemic pesticides, with a high likelihood of residues predominantly in the hulls, the high concentration per unit weight led to the high processing factors [34]. In the case of inner chaff, which refers to the outer hulls inside husked rice, even though the amount of pesticide per unit weight is high, it is determined that the processing factor is less than 1.

### 3.3. Risk Assessment

The average processing factors for flubendiamide were 0.077 and <0.004 for husked rice and polished rice, respectively, while for tebufenozide, they were 0.031 and 0.004, respectively. Since all processing factors were less than 0.1, this indicates that over 90% of the pesticides are reduced when whole grain is milled into husked rice and polished rice. When processed into husked rice, the outer coverings, known as hulls, are removed, while in the case of polished rice, both hulls and inner chaff are removed, leading to lower processing factors in polished rice compared to husked rice. When studying the pesticide reduction results in wheat milling processes similar to the process of removing husks from rice, it was found that azinphos-methyl decreased by 95%, chlorpyrifos decreased by 94%, chlorpyrifos-methyl decreased by 95%, fenitrothion decreased by 93%, malathion decreased by 93%, and trichlorfon decreased by 94% when milling wheat, all indicating a reduction of over 90%, similar to the decrease observed after rice milling processes [35].

The average processing factors for flubendiamide and tebufenozide in husked rice cooked using an electric cooker and a pressure cooker were lower for flubendiamide. When processing into cooked rice, the electric cooker operates at 184 °C, while the pressure cooker operates at 2 atmospheres and 169 °C. Therefore, it was concluded that due to the higher vapor pressure of flubendiamide compared to tebufenozide, more residual pesticide was degraded in flubendiamide, resulting in a smaller processing factor. When processing husked and polished rice into cooked rice and rice cakes, both flubendiamide and tebufenozide had average processing factors of less than 0.5. This means that when processing husked and polished rice into cooked rice and rice cakes consumed by humans, more than 50% of the residue is reduced. According to Ma et al. (2018), similar results were obtained, with a processing factor of 0.27, when polished rice was washed twice and pressure cooked [30].

When processing polished rice, it was not possible to compare the residue concentrations between rice cake and polished rice cooked using an electric cooker, as the residue concentrations in the processed products were less than the limit of quantification. However, significant differences were observed in polished rice cooked using a pressure cooker. This is because the residue concentrations of flubendiamide and tebufenozide in polished rice were <0.01 and 0.06 mg/kg, respectively, and in polished rice cooked using a pressure cooker, they were <0.01 and 0.01 mg/kg, respectively. Since it was possible to calculate the processing factor for tebufenozide but not for flubendiamide due to its residue concentrations being less than the LOQ, a significant difference was observed.

Except for polished rice, husked rice, and rice cake, for processed products, as there are no consumption statistics available in Korea, the estimated daily intake (EDI) was calculated using the average consumption of polished rice cooked by an electric cooker, and a pressure cooker, as well as husked rice cooked by an electric cooker and a pressure cooker, considered as the average consumption of husked rice [36]. The %ADI calculated based on the average intake of Koreans and the average body weight of 60 kg is presented in Table 4. When human exposure to a toxic substance is less than 10% of the ADI, there is generally no need for concern about risk. However, if it exceeds 10%, it requires thorough investigation and strict legal regulation. When it reaches 30% of the ADI level, it is recognized as necessitating a risk warning [37].

The average residue concentrations of tebufenozide in polished rice and husked rice were 0.06 and 0.47 mg/kg, respectively, with husked rice showing a higher level. However, the %ADI for polished rice was approximately six times higher than that for husked rice. This is because the average consumption of polished rice is approximately 24 times higher than that of husked rice. The %ADI for pressure-cooked husked rice, electric-cooked polished rice, and pressure-cooked polished rice were all less than 0.11%, while the residue concentrations in electric-cooked polished rice and rice cake were less than the limit of quantification, making it impossible to calculate %ADI. For flubendiamide, the %ADI for husked rice, pressure-cooked husked rice, and electric-cooked husked rice were all less than 0.10%, while the residue concentrations in the remaining processed products were all less than the limit of quantification, making it impossible to calculate %ADI.

For the processing study, despite applying both flubendiamide and tebufenozide at three times the safe usage standards, the %ADI in all processed products was deemed to be less than 1%, indicating low concern for safety. Even though rice is consumed as a staple food, the %ADI remained less than 1%, and it was deemed to be even lower when the pesticides were applied according to safety usage standards.

## 4. Conclusions

The difference in residue concentrations of the two test pesticides in whole grain was determined by differences in the active ingredient content at the time of pesticide application and the physicochemical characteristics of the pesticides. Both pesticides are non-systemic pesticides, and it was determined that the most influential factor on residue concentrations during the processing process is the milling process, which removes approximately 90% of the pesticide by removing the hulls. Additionally, when polished rice and husked rice are processed into cooked rice and rice cakes, approximately 50% of the pesticide is removed due to washing and heat treatment, resulting in a reduction in residue concentrations even when consumed as cooked rice and rice cakes. For processing study, when both tebufenozide and flubendiamide were sprayed at three-times higher than the safety usage standards, the %ADIs for polished rice, husked rice, cooked rice, and rice cakes were less than 1%, and they are anticipated to be even lower when applied according to safety usage standards. When rice is processed into the form actually consumed as whole grain, most residual pesticides are removed. Therefore, it is determined that conducting processing research to calculate processing factors, along with risk assessment, is necessary to prevent an overestimation of the risk.

## Figures and Tables

**Table 1:** Detailed information of the test pesticides sprayed onto rice.

Site	Pesticide			Application Rate Per Treatment			
		Korean GAP (kg a.i./ha)	3 × Korean GAP (kg a.i./ha)	Treatment	Active Ingredient (kg/ha)	Water (L/ha)	Active Ingredient (kg/hL)
Anseong	Flubendiamide	0.032	0.096	1st 2nd 3rd	0.121 0.120 0.125	2008 2000 2085	0.0075 0.0075 0.0078
	Tebufenozide	0.128	0.384	1st 2nd 3rd	0.504 0.486 0.491	2100 2025 2045	0.0315 0.0304 0.0307

**Table 2:** Multiple reaction monitoring (MRM) conditions of each target pesticide for LC-MS/MS analysis.

Compound	t_R_ (min)	Molecular Weight (g/mole)	Precursor Mass (*m*/*z*)	Quantitation		Confirmation	
				*m*/*z*	CE (V)	*m*/*z*	CE (V)
Flubendiamide	1.8	682.4	680.9 [M − H]^−^	254.1	27	274.0	17
Tebufenozide	1.8	352.5	353.2 [M + H]^+^	133.2	−21	105.2	−40

**Table 3:** Coefficient of determination (R^2^) of test pesticides according to matrices for quantitation in the samples.

Pesticide	Sample	Calibration Point (µg/L)	Linear Range (µg/L)	R^2^
Flubendiamide	Straw	1, 5, 10, 20, 50, 80 and 100	1–100	0.9999
	Hulls		1–100	0.9996
	Inner chaff		1–100	0.9998
	Whole grain	2, 5, 10, 20, 50, 80 and 100	2–100	0.9996
	Husked rice		2–100	0.9998
	Polished rice		2–100	0.9999
	Rice cake		2–100	0.9998
	Husked rice cooked by pressure cooker		2–100	0.9999
	Husked rice cooked by electric cooker		2–100	0.9998
	Polished rice cooked by pressure cooker		2–100	0.9992
	Polished rice cooked by electric cooker		2–100	0.9996
Tebufenozide	Straw	1, 5, 10, 20, 50, 80 and 100	1–100	0.9999
	Hulls		1–100	0.9998
	Inner chaff		1–100	0.9999
	Whole grain	2, 5, 10, 20, 50, 80 and 100	2–100	0.9993
	Husked rice		2–100	0.9994
	Polished rice		2–100	0.9991
	Rice cake		2–100	0.9999
	Husked rice cooked by pressure cooker		2–100	0.9996
	Husked rice cooked by electric cooker		2–100	0.9994
	Polished rice cooked by pressure cooker		2–100	0.9999
	Polished rice cooked by electric cooker		2–100	0.9998

**Table 4:** Risk assessment of flubendiamide and tebufenozide in polished rice and husked rice, and their processed products.

Pesticide	Sample	Mean Residue (mg/kg)	Food Daily Intake ^(b)^ (g)	EDI ^(c)^	ADI ^(e)^	%ADI ^(f)^
				mg/kg b.w./Day		
Flubendiamide	Polished rice	<0.01	117.30	NA	0.017	NA
	Polished rice cooked by electric cooker	<0.01		NA		NA
	Polished rice cooked by pressure cooker	<0.01		NA		NA
	Husked rice	0.19	4.90	1.55 × 10^−5^		0.09
	Husked rice cooked by electric cooker	0.05		4.08 × 10^−6^		0.02
	Husked rice cooked by pressure cooker	0.06		4.90 × 10^−6^		0.03
	Rice cake	<0.01	14.46	NA		NA
Tebufenozide	Polished rice	0.06	117.30	1.17 × 10^−4^	0.02	0.59
	Polished rice cooked by electric cooker	<0.01 ^(a)^		NA ^(d)^		NA
	Polished rice cooked by pressure cooker	0.01		1.96 × 10^−5^		0.10
	Husked rice	0.47	4.90	3.84 × 10^−5^		0.19
	Husked rice cooked by electric cooker	0.23		1.88 × 10^−5^		0.09
	Husked rice cooked by pressure cooker	0.18		1.47 × 10^−5^		0.07
	Rice cake	<0.01	14.46	NA		NA

^(a)^ Less than the limit of quantification (LOQ). ^(b)^ Data from the Korea Health Industry Development Institute (2023), Korea [36]. ^(c)^ Estimated daily intake calculated from the equation of residue (mg/kg) × food daily intake (g). ^(d)^ Not analyzed. ^(e)^ Acceptable daily intake. ^(f)^ (EDI/ADI) × 100.

## Data Availability

The original contributions presented in this study are included in the article. Further inquiries can be directed to the corresponding author.
